# A cross-species approach for the identification of *Drosophila* male sterility genes

**DOI:** 10.1093/g3journal/jkab183

**Published:** 2021-05-29

**Authors:** Kimihide Ibaraki, Mihoko Nakatsuka, Takashi Ohsako, Masahide Watanabe, Yu Miyazaki, Machi Shirakami, Timothy L Karr, Rikako Sanuki, Masatoshi Tomaru, Toshiyuki Takano-Shimizu-Kouno

**Affiliations:** 1 Applied Biology, Graduate School of Science and Technology, Kyoto Institute of Technology, Kyoto 606-8585, Japan; 2 Advanced Technology Center, Kyoto Institute of Technology, Kyoto 606-8585, Japan; 3 Department of Drosophila Genomics and Genetic Resources, Advanced Insect Research Promotion Center, Kyoto Institute of Technology, Kyoto 616-8354, Japan; 4 Mass Spectroscopy Core Facility, Biodesign Institute, Arizona State University, Tempe, AZ 85257-7205, USA; 5 Faculty of Applied Biology, Kyoto Institute of Technology, Kyoto 606-8585, Japan

**Keywords:** human male infertility, male sterility, *Rack1*, cross-species approach, *Drosophila*

## Abstract

Male reproduction encompasses many essential cellular processes and interactions. As a focal point for these events, sperm offer opportunities for advancing our understanding of sexual reproduction at multiple levels during development. Using male sterility genes identified in human, mouse, and fruit fly databases as a starting point, 103 *Drosophila melanogaster* genes were screened for their association with male sterility by tissue-specific RNAi knockdown and CRISPR/Cas9-mediated mutagenesis. This list included 56 genes associated with male infertility in the human databases, but not found in the *Drosophila* database, resulting in the discovery of 63 new genes associated with male fertility in *Drosophila*. The phenotypes identified were categorized into six distinct classes affecting sperm development. Interestingly, the second largest class (Class VI) caused sterility despite apparently normal testis and sperm morphology suggesting that these proteins may have functions in the mature sperm following spermatogenesis. We focused on one such gene, *Rack 1*, and found that it plays an important role in two developmental periods, in early germline cells or germline stem cells and in spermatogenic cells or sperm. Taken together, many genes are yet to be identified and their role in male reproduction, especially after ejaculation, remains to be elucidated in *Drosophila*, where a wealth of data from human and other model organisms would be useful.

## Introduction

Like many animals, male reproduction of *Drosophila* encompasses many different cellular processes such as stem-cell division and maintenance, spermatogonial mitotic divisions, mitosis-to-meiosis transition, mitochondrial transformation, compaction of sperm chromatin, spermatid elongation, maturation, and storage (Fuller 1993). Moreover, ejaculated sperm and seminal fluid proteins combine and mix during copulation, travel to the female reproductive tract, and are stored in the specialized storage organs, a seminal receptacle, and paired spermathecae. Sperm storage and utilization are highly efficient processes with approximately 500 sperm in storage (Manier *et al.* 2010); the majority (∼300) were used within a week under normal laboratory conditions (Manning 1967; Herndon and Wolfner 1995; Kubli 1996, 2003). Sperm competition is also well documented in *Drosophila* and many other species (Harshman and Clark 1998; Price *et al.* 1999; Chen *et al.* 2019) in cases where sperm from different males are present (Milkman and Zeitler 1974; Griffiths *et al.* 1982; Harshman and Clark 1998). Thus, male reproduction encompasses interactions at many different levels including (i) protein, *e.g.*, sperm-by-seminal fluid proteins (Wolfner 1997) and paternal-by-maternal molecular interactions (Loppin *et al.* 2005); and (ii) cellular, *e.g.*, sperm-by-egg (Karr 1991; Karr and Pitnick 1996; Snook and Karr 1998; Perotti *et al.* 2001), stem cell-by-spermatogonia (Brawley and Matunis 2004), niche-by-stem cell (Fuchs *et al.* 2004; Moore and Lemischka 2006; Morrison and Spradling 2008), and germ cell-by-somatic cyst cell (Leatherman and DiNardo 2010); (iii) tissue and organ levels, *e.g.*, sperm-by-female reproductive tract (Miller and Pitnick 2002), and sperm-by-sperm (sperm competition) interactions. As a focal point for these events, a molecular genetic study of sperm offers unique opportunities for advancing our understanding of sexual reproduction at multiple levels during development.

Despite such an important model system, male reproduction seems to be less studied and less well characterized. An indication of this comes from a database study of human and model organisms. The human disease databases, the Online Mendelian Inheritance in Man (OMIM, https://www.ncbi.nlm.nih.gov/omim) and GeneCards (https://www.genecards.org), together with list more than 1000 genes that are associated with male infertility. However, many of their orthologs are not found in male infertility or sterility gene lists extracted from the mouse and fly databases (Mouse Genome Informatics, MGI, http://www.informatics.jax.org; International Mouse Phenotyping Consortium, IMPC, https://www.mousephenotype.org; and FlyBase, https://flybase.org). The low level of overlap among the three male infertility and sterility gene lists suggests that these gene lists are far from complete and that many genes remain to be identified in all three species. We therefore examined and compared the human male infertility gene list with *Drosophila* without regard to whether or not the *Drosophila* genes were annotated with male sterility. The resulting list included 103 fly genes, including 56 genes associated with male infertility in the human databases but not annotated as such in FlyBase. The function of these genes in sperm development was then screened using tissue-specific RNAi knockdown (a total of 155 RNAi stocks), finding 63 new genes that are likely associated with male sterility in *Drosophila*. This finding not only supports the hypothesis that many genes remain to be identified in both human and fly, but also highlighted the potential advantage of cross-species study, particularly using human data.

Of the 63 new genes identified in our screen, *Rack1*, a gene involved in protein kinase C signal transduction, was chosen for additional study. As a Class VI phenotype gene, RNAi knockdown of *Rack1* resulted in complete sterility without obvious morphological changes in the testis and sperm. This class of mutants are particular intriguing because such genes have rarely been reported despite the expectation that the many modifications of sperm occur during maturation and travel into, and through, the female reproductive tract (Castrillon *et al.* 1993; Ohsako and Yamamoto 2011; Skerget *et al.* 2015). We confirmed the association with male sterility by CRISPR/Cas9-mediated mutagenesis and then found that *Rack1* plays an important role in two developmental periods, in early germline cells or germline stem cells and in spermatogenic cells or sperm. Finally, we also show that the human *RACK1* gene can substitute at least for the latter function of the fly *Rack1*, further supporting the similarity of the mechanisms of reproduction in the two species.

## Materials and methods

### *Drosophila* strains

To drive expression of UAS constructs in testes, we used two germline and two soma *GAL4* drivers: *nos-GAL4* (KYOTO Stock Center, DGRC 107955), *bam-GAL4* (provided by Dennis M. McKearin), *ptc-GAL4* (DGRC 106629), and *upd-GAL4* (provided by Ting Xie). RNAi strains used in this study are listed in Reagents table. We also used two *UAS-Cas9.P* strains to apply the CRISPR/Cas9 system (Bloomington Drosophila Stock Center, BDSC 54594 and BDSC 54592), and *ProtB-GFP* strain (DGRC 109643) to visualize sperm. We also used Oregon R (DGRC 105669) and a highly inbred *y w* (TT16, Tanaka *et al.* 2014) stocks as controls.

### Construction of UAS-ORF and UAS-sgRNA clones and strains

Fly *Rack1* and human *RACK1* open reading frames (ORFs) were amplified by PCR using the RE74715 cDNA gold clone obtained from Drosophila Genomics Resource Center and the 3455986 MGC clone from DNAFORM, respectively. They were first cloned into the pCR8/GW/TOPO or pENTR/D-TOPO (Invitrogen) and then into pUASg_attB vector (Bischof *et al.* 2013). The plasmid DNA was injected into embryos of *y M{vas-int.Dm}ZH-2A w; PBac{y[+]-attP-3B}VK00033* (DGRC 130448) and *y v P{nos-phiC31\int.NLS}X; P{y[+t7.7]-CaryP}attP40* (BDSC 25709).

To generate a single-guide RNAs (sgRNAs) construct, we first determined *Rack1* sequences of strains used for CRISPR/Cas9 mutagenesis and then designed sequences of sgRNAs that targeted exon-intron boundaries, one in that of first exon/first intron and the other in that of first intron/second exon, by using CRISPR direct (http://crispr.dbcls.jp; Naito *et al.* 2015). These two targeted sequences were then cloned into a single pCFD6 vector by PCR with Q5 High-Fidelity 2X Master Mix (NEB) and NEBuilder HiFi DNA Assembly (NEB), resulting in a construct of *UAS-(DmtRNAGly)-(gRNA-Rack1[1])-(OstRNAGly)-(gRNA-Rack1[2])-(OstRNAGly)-terminator* (Port and Bullock 2016). The construct was then injected into embryos of *y M{vas-int.Dm}ZH-2A w; PBac{attP-3B}VK00033*. Primers (gR_CG7111_F1 and gR_CG7111_R1) used for the PCR amplification are given in Reagents table and the CRISPR/Cas9 target sequences are TGGCGATTACTTACCACGGG and CAGACAAGACCCTGATCGTG.

### RNAi screening

We crossed two (2- or 3-day-old) females of the *GAL4* driver strains to two males of *UAS-RNAi* strains at 25° for a few days and then incubated the vials at 27° until adult offspring emerged. F1 virgin males (RNAi males) were collected and aged at 25°, and all subsequent crosses were also done at 25°. At the same time, five pairs of *y w* strain were placed in a vial, and discarded 2 days later. The F1 virgin females were collected at 25°. For fertility assessment, a single 2- or 3-day-old RNAi male was crossed to a single *y w* female. They were transferred to a new vial 4 or 5 days later, and discarded on the ninth day after cross. As controls, we counted the number of offspring of single *y w* females crossed with single males of the following six strains: Oregon R [the number of replications (*n*) = 8, mean ± standard deviation = 92.0 ± 10.4, minimum—maximum = 78–112], *y w* (*n *=* *8, 65.0 ± 9.1, 53–79), *nos-GAL4* (*n *=* *8, 98 ± 6.7, 89–107), *bam-GAL4* (*n *=* *5, 84.2 ± 6.8, 75–91), *upd-GAL4* (*n *=* *8, 88.0 ± 8.6, 74–99), and *ptc-GAL4* (*n *=* *8, 98.0 ± 6.7, 87–109). From these data, we chose a cut-off point of less than 30 offspring for semi-sterile males, which was distinguished from sterile ones that produced no offspring. We dissected 2- or 3-day-old virgin males and assessed visually testis morphology by light microscopy, the presence of sperm in the seminal vesicles, and sperm motility. We then classified 110 sterile or semi-sterile males into six classes following the convention of previous studies (Castrillon *et al.* 1993; Wakimoto *et al.* 2004): Class I, proliferation- or growth-phase defect (reduced cell number or thin testis); Class II, meiotic entry or meiotic division defect (no onion-stage spermatid or no early elongated spermatid); Class III, elongation, coiling, or individualization defect (no accumulation of coiling spermatids); Class IV, spermatid-release defect (accumulation of coiling spermatids in the distal end of testis); Class V, no or very few mature sperm in the seminal vesicle despite apparently normal spermatogenesis; Class VI, apparently normal testis. In addition, E refers to no sperm in seminal vesicles in Supplementary Table S5.

### Fertility test

Fertility of *Rack1* RNAi knockdown males was measured by the number of offspring and hatchability of eggs produced by single females mated with single males in question. Females of the *GAL4* driver strains were crossed to males of *UAS-RNAi[Rack1]* strains (*RNAi-KK109073*, VDRC 104470, and *RNAi-TRiP.GL00637*, BDSC 38198) and transferred to new vials every 3 days. But, unlike the RNAi screening experiments, all tested males were raised at 25° during the entire period of experiments. Three- to five-day-old virgin F1 males were individually crossed to *y w* single females. Females copulated 15 minutes or more were isolated and then allowed to lay eggs in single vials for 3 days to count the number of offspring. The parental females were placed to egg counting chamber with a 20-well grape-juice-agar plate for 1 day and transferred to new plates twice (in total, three plates for each female) to assess egg hatchability. One and two days after transfer, we counted the number of hatched and unhatched eggs. For rescue experiments, *bam*-*GAL4* females were crossed to males of *RNAi-KK109073; UAS-dRack1* or *RNAi-KK109073; UAS-hRACK1* strains.

For CRISPR/Cas9-mediated mutagenesis, females carrying *GAL4* and *UAS-Cas9.P* were crossed to males of the *UAS-sgRNAs* strain and we assessed male fertility in the same way.

### Sperm count in female reproductive organs

We crossed *bam-GAL4*; *ProtB-GFP* females to *RNAi-KK109073* males and then obtained F1 males as in the RNAi knockdown experiments. Virgin F1 males as well as *bam-GAL4*; *ProtB-GFP* males as a control were aged for 3 to 5 days and then individually crossed to *y w* females. Females copulated 15 minutes or more were dissected and fixed at 1 hour after the end of copulation. We counted the number of sperm in the reproductive organs by eyes.

### Data availability

All database search results are presented in Supplementary Tables S2 –S4 and the RNAi screening results in Supplementary Table S5. All strains used in this study are included in the Reagent Table and the newly generated *UAS-dRack1* and *UAS-hRACK1* strains are available from KYOTO Stock Center.

Supplementary material is available at *G3* online.

## Results

### RNAi screening

In total, 1390 human genes associated with male infertility were identified in the Online Mendelian Inheritance in Man (OMIM, https://www.ncbi.nlm.nih.gov/omim, as of March 18, 2020) and GeneCards (https://www.genecards.org, as of March 14, 2020) databases (Table 1 and Supplementary Table S2). However, many of their orthologs were not found in male infertility or sterility gene lists (Supplementary Tables S3 and S4) extracted from the mouse and fly databases (Mouse Genome Informatics, MGI, http://www.informatics.jax.org; International Mouse Phenotyping Consortium, IMPC, https://www.mousephenotype.org; and FlyBase, https://flybase.org). Indeed, orthologous relationships exist only between 278 (20.0%) out of the 1390 human genes and 272 (26.9%) of 1013 mouse genes and between 120 (8.6%) of the human genes and 88 (21.5%) of 410 fly genes. The fraction of common genes were even lower between the two model organisms, 8.3% of the mouse genes and 17.8% of the fly genes. The low similarities among the three male infertility and sterility gene lists are partly due to different methodologies adopted by the respective scientific communities for gene discovery; in particular, the history and phenotypic description of human male infertility quite differ from those of the model organisms. The main causes of human male infertility include varicocele, hypogonadism, urogenital infection, immune system factors, sexual factors, and systemic disease (Poongothai *et al.* 2009; Jungwirth *et al.* 2012). They are not necessarily directly related to sperm viability and functions; variants affecting such processes are potentially included in the human infertility gene list. It is very likely that these gene lists are far from complete and that many genes remain to be identified in all three species. Given this notion of the incompleteness of these lists, the human male infertility gene list could be used to identify new male sterility genes of *Drosophila*.

**Table 1:** Male infertility protein-coding genes in human, mouse, and fly databases

Species	Source	Search date	Search term	Number of genes
Human	GeneCards	March 14, 2020	“male infertility”	1,365
—	OMIM^*a*^	March 18, 2020	“male infertility”	120
—	Sum	—	—	**1,390**
—	—	—	1,378 (99.1%) have known mouse orthologs	
—	—	—	1,102 (79.3%) have known fly orthologs	
Mouse	MGI^*b*^	March 14, 2020	“male infertility”	710
—	IMPC^*c*^	March 14, 2020	“male infertility”	489
—	Sum	—	—	**1,013**
—	—	—	899 (88.7%) have known fly orthologs	
Fly	FlyBase	March 14, 2020	“male sterility”	410

We searched the databases for “male infertility” human and mouse genes and “male sterility” fly genes and extracted only protein-coding genes (Supplementary Tables S2–S4).

a OMIM: Online Mendelian Inheritance in Man.

b MGI: Mouse Genome Informatics.

c IMPC: International Mouse Phenotyping Consortium.

Here, we tested this possibility by RNAi screening of 103 fly genes (Supplementary Table S1) including 56 ones associated with male infertility in the human databases but not in the FlyBase. We performed tissue-specific knockdown of those fly genes (155 RNAi stocks) by using two germline and two somatic *GAL4* drivers: *nos-GAL4* for germline cells from embryonic stage 9 onward, germline stem cells (GSC), and spermatogonia, *bam-GAL4* for late spermatogonia and early spermatocytes, *ptc-GAL4* for cyst stem cells and cyst cells, and *upd-GAL4* for hub cells. The results are given in Supplementary Table S5, in which 61 genes were found to cause male sterility or semi-sterility when combined with *bam-GAL4*, 10 genes with *nos-GAL4*, 26 genes with *ptc-GAL4*, and 22 with *upd-GAL4*. In sum, RNAi knockdown males of 31 and 44 genes caused male sterility and semi-sterility, respectively (see Materials and Methods for their definitions). Out of 13 genes associated with male sterility in FlyBase (Supplementary Table S1), 12 were confirmed in this screen (10 male sterile and 2 semi-sterile genes); the only exception is *Ecdysone receptor* (*EcR*, CG1765). A single PZ insertion in an intron of *EcR* is reported to cause male sterility, but, to our best knowledge, this has not been verified yet (Spradling *et al.* 1999). Therefore, our screening approach identified known genes associated with male sterility while uncovering additional genes previously not known to be associated with this phenotype. By using the orthologs of the human genes associated with male infertility, we found 63 new genes that were likely involved in male fertility in *Drosophila*. This finding supports our notion that many genes remain to be identified in both human and fly.

While the phenotypes associated with reduced male fertility largely varied among both genes and drivers (Supplementary Table S5), the largest were Class I (proliferation- or growth-phase defect with 31 appearances) and Class VI (apparently normal testis with 28 appearances). The *bam-GAL4* driver phenotypes ranged evenly across all six classes, while *upd*- and *ptc*-GAL4 driver combinations tended to concentrate on Classes I and VI. Class VI includes six genes for which RNAi knockdown caused complete male sterility (three with *bam-GAL4*, one with *nos-GAL4*, and two with *ptc-GAL4*, Supplementary Table S5).

### *Rack1* is required for male fertility in two developmental periods

*Receptor of activated protein kinase C 1* (*Rack1*) is a *Drosophila* ortholog of human *RACK1* gene, which encodes a scaffolding protein involved in recruitment, assembly, and regulation of a variety of signaling molecules (GeneCards, www.genecards.org, Stelzer *et al*. 2016). RACK1 protein is identified as one of epididymal sperm-located proteins (Li *et al.* 2010). In addition, *RACK1* is suggested to be associated with Noonan Syndrome 1, which could cause male infertility (MalaCards, www.malacards.org, Rappaport *et al.* 2017). Mutants of the essential *Drosophila Rack1* gene are known to cause female sterility (Kadrmas *et al.* 2007), but its association with male sterility has not yet been known. Indeed, the expression level of *Rack1* is very low in adult males than in embryos, larvae, and adult females (Kadrmas *et al.* 2007). The present RNAi phenotypes with the *bam-GAL4* driver are the first indication of an involvement of the *Rack1* with male fertility. Here, we added tissue-specific CRISPR/Cas9-mediated mutagenesis to the RNAi knockdown experiments and characterized the roles of the *Rack1* in male fertility in more detail.

**Figure 1:**
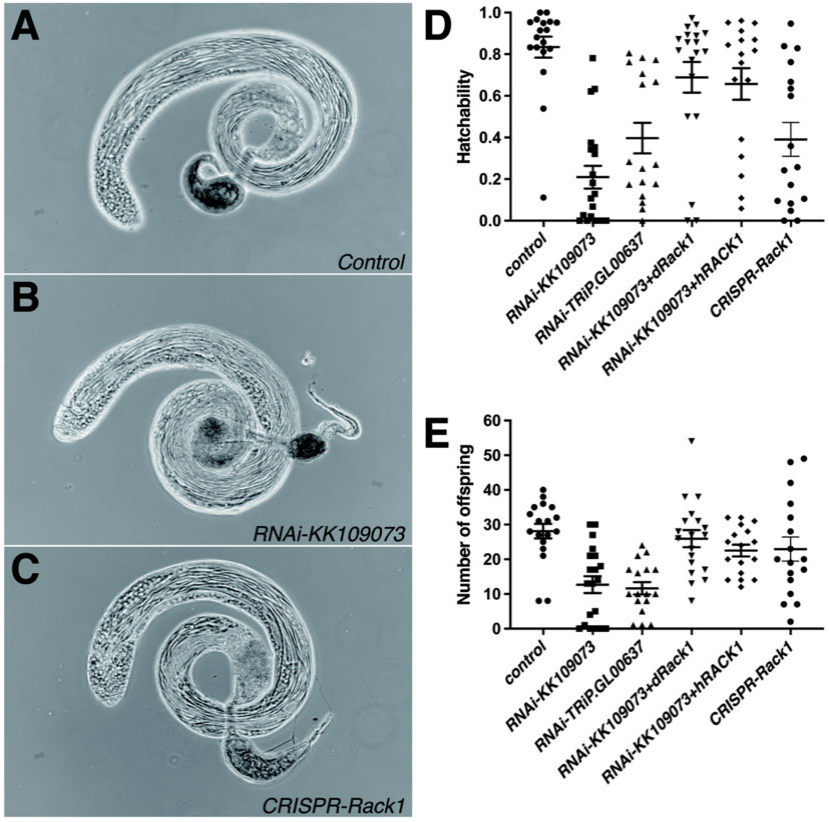
Reduced fertility of *bam-GAL4*-driven *Rack1* RNAi-knockdown and CRISPR/Cas9-mutagenized males without obvious morphological changes and its rescue by concomitant expression of *Drosophila Rack1* or human *RACK1* ORF. Testes of *bam-GAL4*/Y; *CyO*/+ (control) (A), *bam-GAL4*/Y; *RNAi-KK109073*/+ (B), and *bam-GAL4*/Y*; UAS-Cas9.P*/*UAS-sgRNAs-Rack1* (C) males. Hatchability of eggs (D) and the number of offspring (E) produced by single females mated with treated males with standard error of the mean. The genotypes of males were *bam-GAL4*/Y; *CyO*/+ (control, *bam*>+), *bam-GAL4*/Y; *RNAi-[Rack1]*/+ (*bam*>*RNAi-KK109073* and *bam*>*RNAi-TRiP.GL00637*), *bam-GAL4*/Y; *RNAi-KK109073*/+; *UAS-dRack1* (or *UAS-hRACK1)*/+ (*bam*>*RNAi-KK109073*+*dRack1* and *bam*>*RNAi-KK109073*+*hRACK1*), and *bam-GAL4*/Y*; UAS-Cas9.P*/*UAS-sgRNAs-Rack1* (*bam*>*CRISPR-Rack1*). Egg hatchability of *bam*>*RNAi-KK109073*, *bam*>*RNAi-TRiP.GL00637*, and *bam*>*CRISPR*-*Rack1* males was significantly lower than that of control, and that of *bam*>*RNAi-KK109073*+*dRack1* and *bam*>*RNAi-KK109073*+*hRACK1* was significantly higher than that of *bam*>*RNAi-KK109073* (all *P *<* *0.0001, Mann–Whitney *U*-test). The number of offspring produced by *bam*>*RNAi-KK109073* and *bam*>*RNAi-TRiP.GL00637* was significantly smaller than that of the control (both *P *<* *0.0001, Mann–Whitney *U*-test), while that of *bam*>*RNAi-KK109073*+*dRack1* (*P *<* *0.001, Mann–Whitney *U*-test) and *bam*>*RNAi-KK109073*+*hRACK1* (*P *<* *0.01, Mann–Whitney *U*-test) was significantly larger than that of *bam*>*RNAi-KK109073*.

On the basis of the present RNAi screening results with the *bam-GAL4* driver, *Rack1* is one of the three genes that exhibited the Class-VI sterile phenotype: male sterility without obvious changes in the testis and sperm morphology (Figure 1, B, D, and E). The Class VI mutants are particularly intriguing because such genes remain rare despite the expectation that many changes occur in sperm during maturation and traveling in female reproductive tract (Castrillon *et al.* 1993; Ohsako and Yamamoto 2011; Skerget *et al.* 2015). To further characterize the roles of the *Rack1* in male fertility, we first confirmed the reduced fertility of *Rack1* RNAi knockdown males by crossing additional RNAi line (TRiP.GL00637) with the *bam-GAL4* driver (Figure 1, D and E). Eggs laid by females mated with these RNAi knockdown males had significantly reduced hatchability. In addition to the *Rack1* RNAi construct, concomitant expression of *Drosophila Rack1* (*dRack1*) open reading frame (ORF) restored egg hatchability, implying that *Rack1* is indeed required for normal male fertility (Figure 1, D and E).

To further understand the cause of the reduced fertility of *Rack1* RNAi knockdown males, we examined sperm storage in seminal receptacle and a pair of spermathecae at 1 hour after copulation by using a *ProtB-GFP* marker that produces highly fluorescent sperm nuclei (Manier *et al.* 2010). While there was no difference in the total number of sperm in the uterus and sperm storage organs between the two types of males, the number of sperm in the female’s sperm storage organs was significantly reduced for the *Rack1* RNAi knockdown males as compared to the control males (Table 2 and Supplementary Figure S1). These results suggested that the sperm were immature or not fully activated. Indeed, while sperm of the control males were localized at the anterior uterus where the sperm storage organs are located, sperm of the *Rack1* RNAi males remained scattered throughout the uterus (Supplementary Figure S2).

**Table 2:** The number of sperm stored in female’s sperm storage organs

Male	Uterus	Spermathecae	Seminal receptacle	Sum
*bam-GAL4*/Y*; ProtB-GFP*	990.4 ± 72.4 (547–1,244)	295.8 ± 9.3 (247–329)	637.9 ± 24.9 (544–756)	**1,924.1 ± 87.7 (1,385–2,258)**
*bam-GAL4*/Y; *RNAi-KK109073*/+; *ProtB-GFP*/+	1,419.5 ± 105.1 (1,127–1,975)*	158.5 ± 36.6 (14–361)**	201.0 ± 59.2 (3–538)***	**1,779.0 ± 80.3 (1,477–2,129)**

Virgin males were individually mated with single females and the number of sperm was counted at 1 hour after copulation. For both males, the sample size was ten. The numbers given are means ± standard errors of the means with minimum and maximum numbers in parentheses. Mann–Whitney *U*-test was performed to test differences in sperm number between the two groups (*P <* *0.05, **P <* *0.01, and ***P <* *0.002).

Tissue-specific CRISPR/Cas9-mediated mutagenesis with the *bam-GAL4* driver and two gRNAs targeting *Rack1* also significantly reduced hatchability (Figure 1). This again suggested that normal transcription of *Rack1* gene in late spermatogonia or early spermatocytes is required for male fertility.

**Figure 2:**
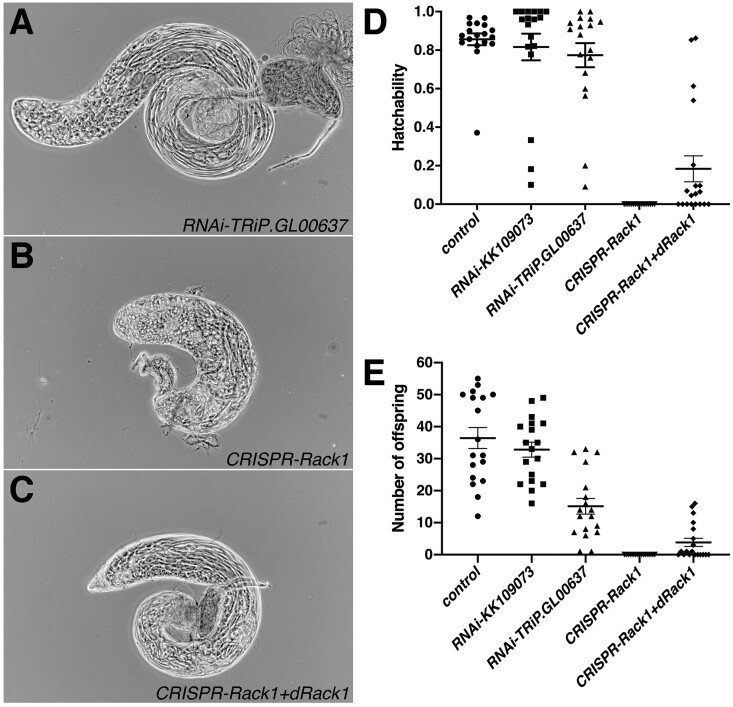
No obvious phenotype in *nos-GAL4*-driven *Rack1* RNAi-knockdown males, but testis underdevelopment and disruption of spermatogenesis of CRISPR/Cas9-mutagenized males. Testes of *RNAi-TRiP.GL00637*/+; *nos-GAL4*/+ (A), *UAS-Cas9.P*/+; *nos-GAL4*/*UAS-sgRNAs-Rack1* (B), and *UAS-Cas9.P*/*UAS-dRack1*; *nos-GAL4*/*UAS-sgRNAs-Rack1* (C) males. Hatchability of eggs (D) and the number offspring (E) produced by single females mated with treated males with standard error of the mean. The genotypes of males were *CyO*/+; *nos-GAL4*/+ (control), *RNAi-KK109073*/+; *nos-GAL4*/+ (*RNAi-KK109073*), *RNAi-TRiP.GL00637*/+; *nos-GAL4*/+ (*RNAi-TRiP.GL00637*), *UAS-Cas9.P*/+; *nos-GAL4*/*UAS-sgRNAs-Rack1* (CRISPR-*Rack1*), and *UAS-Cas9.P*/*UAS-dRack1*; *nos-GAL4*/*UAS-sgRNAs-Rack1* (CRISPR-*Rack1*+*dRack1*) males. There was no significant difference in hatchability and the number of offspring between the control and RNAi knockdown males, except for the number of offspring between the control and *RNAi-TRiP.GL00637* males (*P *<* *0.0001, Mann–Whitney *U*-test). The CRISPR/Cas9-mediated mutagenized males were completely sterile that was partially rescued by concomitant expression of *Drosophila Rack1*.

Consistent with the first RNAi screening result, we detected neither a significant reduction in egg hatchability and the number of offspring nor morphological changes in testes and sperm when using the second RNAi and *nos-GAL4* driver lines (Figure 2, A, D, and E). However, CRISPR/Cas9-mediated mutagenesis caused marked underdevelopment of testes and spermatogenesis was severely disrupted, although the testes still seem to contain some cells at various stages of differentiation including spermatocytes and spermatids (Figure 2B). In any case, *Rack1* mutagenized males were completely sterile (Figure 2, D, and E). The sterility was partially rescued by expression of *Rack1* (Figure 2, C–E), in which two sgRNA were designed to target exon-intron boundaries and then exogenous *Rack1* ORF was expected not to be affected by the CRISPR/Cas9 mutagenesis. Taken together, we suggest that *Rack1* also plays an essential role in germline cells from embryonic stage 9 onward or germline stem cells.

### Human *RACK1* can replace *Drosophila Rack1* during spermatogenesis

Like *Drosophila Rack1*, overexpression of human *RACK1* ORF significantly increased the hatchability of eggs laid by females mated with the RNAi-treated males (Figure 1, D and E) and there was no difference in the effect between the *Drosophila* and human ORFs.

## Discussion

Mainly on the basis of human database information combined with mouse and *Drosophila*, we identified 63 new genes that were likely associated with male fertility in *Drosophila*, suggesting the similarity at the molecular level of male reproduction between human and fly. We propose that the results obtained here are representative of, and applicable to other, human male infertility genes and that many genes remain to be identified in both species. An obvious question immediately arises: how many genes are involved in male reproduction in general? Powerful proteomic analyses of *Drosophila* sperm and testes identified more than 1000 protein genes (Dorus *et al.* 2006; Takemori and Yamamoto 2009; Wasbrough *et al.* 2010) and proteomes of seminal fluid proteins independently identified more than 100 protein genes (Walker *et al.* 2006; Findlay *et al.* 2008; see also Ravi Ram and Wolfner 2007). In human, more than 2000 proteins are listed as male proteins such as testis, spermatozoon, and seminal vesicle proteins (Wilhelm *et al.* 2014; Uhlén *et al.* 2015); in mouse, 1766 proteins are identified from sperm collected from the epididymis (Skerget *et al.* 2015). More recently, Gärtner *et al.* (2019) studied testes proteomes of *Drosophila* larvae, pupae, and adults separately and identified, in total, 6171 proteins. On the other hand, transcriptional analyses unveil the highly complex testis transcriptome, with more than 10,000 genes expressed in both *Drosophila* (Parisi *et al.* 2004; Vibranovski *et al.* 2009; Graveley *et al.* 2011; Vedelek *et al.* 2018) and mammals (Ramsköld *et al.* 2009; Soumillon *et al.* 2013). In sum, it is conceivable that, although the exact number is not known, much more protein genes remain to be identified.

If many genes are involved in male sterility, why did they fail to be identified? Like other developmental processes, genetic screens in *Drosophila* (Hackstein 1991; Castrillon *et al.* 1993; Wakimoto *et al.* 2004) have been productive. Especially, Wakimoto *et al.* (2004) identified 2131 male sterile lines from a collection of lines carrying chemically mutagenized chromosomes; to our best knowledge, many of them have not been molecularly characterized so far. Recently, in the same line of our study, Yu *et al.* (2015) have studied 22 *Drosophila* genes based on human genome-wide association study data for nonobstructive azoospermia by RNAi and successfully identified 7 genes essential for male fertility. Wen *et al.* (2016) also screened 105 long noncoding RNAs by CRISPR/Cas9 system and found 33 genes required for normal male fertility. A shortage of large-scale studies exploring male sterile genes at the molecular level is still a reason for the incompleteness of the male sterility gene list. Despite an important model system, many genes are yet to be identified and their roles in male reproduction remain to be elucidated.

To validate the RNAi screening results, we performed a further analysis of *Rack1*, which is one of the six genes exhibited the Class-VI sterile phenotype, and obtained evidence that *Rack1* is indeed essential for male fertility. Downregulation of *Rack1* under the control of *bam-GAL4* reduced fertility in the two RNAi lines studied. In contrast, when combined with the *nos-GAL4* driver, both lines did not produce any phenotype. The two drivers differ in the timing of expression. The *nos-GAL4* driver induces target expression in germline cells from embryonic stage 9 onward and germline stem cells (Van Doren *et al.* 1998), while the *bam-GAL4* driver does not induce expression in germline stem cells, but does in late spermatogonia and early spermatocytes (Chen and McKearin 2003). Thus, one explanation for the contrasting results between the two drivers is that the *Rack1* expression level required in late spermatogonia and early spermatocytes may be higher than that in germ cells in the earlier stages. This is not surprising because the time window for transcription in the former cells is much shorter than that in germline stem cells (Olivieri and Olivieri 1965; see also Barreau *et al.* 2008). Alternatively, although not mutually exclusive, the *Rack1* knockdown efficiency by the *nos-GAL4* driver may not be sufficiently high compared to the *bam-GAL4* driver. Given the present results, the former explanation is more likely. The phenotypes were consistent across the two independent RNAi lines and the reduced hatchability of eggs laid by females mated with *bam*>*Rack1* RNAi-knockdown males was largely rescued by concomitant expression of the *UAS-Rack1* transgene. What is more, CRISPR/Cas9-mediated mutagenesis combined with the *bam-GAL4* driver also resulted in reduced hatchability. These results indicate a requirement of *Rack1* transcription in developing spermatogenic cells. On the other hand, when we combined the CRISPR/Cas9 system with the *nos-GAL4* driver, the resultant males were characterized by marked underdevelopment of testes and disruption of spermatogenesis (Figure 2C), suggesting a requirement of *Rack1* in the earlier stages, namely in early germline cells or germline stem cells or both. Thus, there seem to be two developmental periods, in which *Rack1* transcription is required for male fertility.

*Rack1* and its orthologs are known to be required for migratory and neuronal cells. The *Drosophila Rack1* gene is essential for migration and cluster cohesion of border cells, a group of migratory follicle cells, possibly by regulating cell-cell adhesion between border cells or between border cells and nurse cells (Luo *et al.* 2015). The *Caenorhabditis elegans rack-1* gene is required for lamellipodia and filopodia formation, axon pathfinding, and migration of the gonadal distal tip cells (Demarco and Lundquist 2010). In addition, mouse RACK1 protein is localized to, and required for the formation of, point contacts in growth cones, which are adhesion sites linking the actin network within growth cones to the extracellular matrix and local translation sites (Kershner and Welshhans 2017). *Rack1* may also play a role in cell-cell adhesion or contact-dependent signaling during spermatogenesis and sperm maturation; however, at present, the exact biological functions of RACK1 protein remain to be explored and are the subject of future investigation.

In this study, Class VI phenotype observed in *bam>Rack1* RNAi constitutes the second largest class in the present screen, although such mutants remain rare (Castrillon *et al.* 1993; Ohsako and Yamamoto 2011). This may reflect the general nature of hypomorphic mutations and we could see earlier phenotypes by gene disruption. Alternatively, these gene products may be required specifically for sperm maturation or post-ejaculation function. While we cannot rule out the former, the latter may be applied to several genes including *Rack1*. Indeed, early stages of spermatogenesis such as stem-cell maintenance and meiotic division have been extensively studied, but not the late stages so far; therefore, much more genes could be involved in sperm maturation and post-ejaculation function.

In this study, we showed that the human data resulted mostly from genome-wide association study (GWAS) and omics study are effectively evaluated in fruit fly. The availability of many types of comprehensive and integrated databases (Brody 2019) makes such cross-species studies easier to perform. Together with them, we will soon have a more comprehensive understanding of male reproduction and therefore of male-female interactions before and after fertilization.

## Supplementary Material

jkab183_Supplementary_Data
