# Supplementary material for: A cross-species approach for the identification of Drosophila male sterility genes
Source: G3 (Bethesda). 2021 May 29;11(8):jkab183. doi: 10.1093/g3journal/jkab183 (PMC8496277; doi:10.1093/g3journal/jkab183)
Supplement: jkab183_Supplementary_Data [file jkab183_supplementary_data.zip › jkab183-suppl_data/GENETICS-G3-2021-402310-s09.docx]

Supplemental Figure LEGENDS

**Supplemental Figure 1** Sperm in female sperm storage organs, seminal receptacle and spermatheca. Seminal receptacle (A and B) and spermathecae (C and D) were dissected together with uterus at 1 h after the end of copulation and observed under a fluorescent microscope (Nikon Eclipse 80i, Japan). (A and C) are storage organs of females mated with control males (*bam-GAL4*/Y; *ProtB-GFP*), (B and D) are those of females mated with the *Rack1* RNAi males (*bam-GAL4*/Y; *RNAi-KK109073*/+; *ProtB3-GFP*/+). GFP-labeled sperm heads are shown in green with auto-fluorescence under UV illumination in magenta in left panels and in white in right panels.

**Supplemental Figure 2** Sperm in uterus at 1 h after the end of copulation. (A) shows four uteruses of females mated with control males (*bam-GAL4*/Y; *ProtB-GFP*) and (B) is those of females mated with the *Rack1* RNAi males (*bam-GAL4*/Y; *RNAi- KK109073*/+; *ProtB-GFP*/+). GFP-labeled sperm heads are shown in green and autofluorescence under UV illumination in magenta shows mating plug. All uteruses are oriented anterior to the left.
