# Supplementary figures and images for: A cross-species approach for the identification of Drosophila male sterility genes
Source: G3 (Bethesda). 2021 May 29;11(8):jkab183. doi: 10.1093/g3journal/jkab183 (PMC8496277; doi:10.1093/g3journal/jkab183)

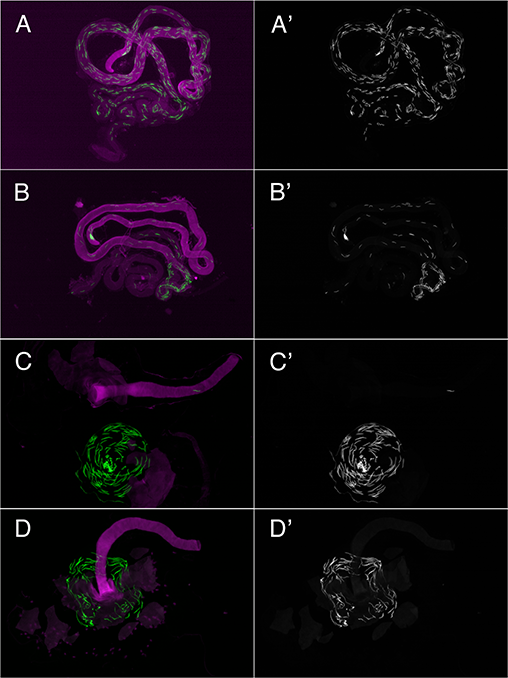

Supplement: jkab183_Supplementary_Data [file jkab183_supplementary_data.zip › jkab183-suppl_data/GENETICS-G3-2021-402310-s01.tif]

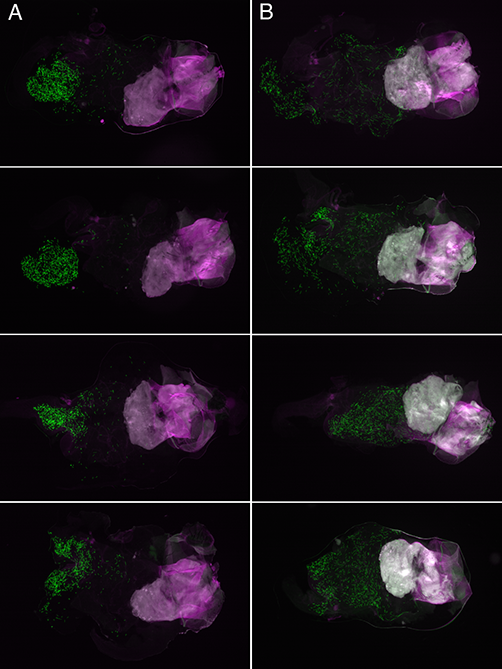

Supplement: jkab183_Supplementary_Data [file jkab183_supplementary_data.zip › jkab183-suppl_data/GENETICS-G3-2021-402310-s02.tif]
